# Effectiveness of distance-based suicide interventions: multi-level meta-analysis and systematic review

**DOI:** 10.1192/bjo.2022.526

**Published:** 2022-07-21

**Authors:** Jim Schmeckenbecher, Katrin Rattner, Robert J. Cramer, Paul L. Plener, Anna Baran, Nestor D. Kapusta

**Affiliations:** Department of Psychoanalysis and Psychotherapy, Medical University Vienna, Austria; Clinic for Psychiatry and Psychotherapy, kbo-Inn-Salzach-Klinikum, Freilassing, Germany; Department of Public Health Sciences, University of North Carolina at Charlotte, North Carolina, USA; Department of Child and Adolescent Psychiatry, Medical University Vienna, Austria; and Department of Child and Adolescent Psychiatry and Psychotherapy, University of Ulm, Germany; Department of Medicine and Optometry, Faculty of Health and Life Sciences, Linnaeus University, Sweden; and Department of Psychiatry, Blekinge Hospital, Sweden

**Keywords:** Suicide, suicidal behaviour, meta-analysis, distance-based intervention, psychosocial interventions

## Abstract

**Background:**

The use of distance-based interventions (DBIs) to reduce suicidal ideation and behaviours are an increasingly relevant form of intervention. DBIs are more affordable, scalable and available than traditional face-to-face interventions, helping to narrow the gap between needed and provided care.

**Aims:**

To evaluate the overall effectiveness of DBIs against suicidal ideation and behaviours.

**Method:**

We systematically searched Web of Science, Scopus and PubMed for all DBIs primarily aimed at reducing suicidal ideation and behaviours. Data were analysed with a robust variance estimation corrected, multi-level meta-analysis.

**Results:**

We found 38 studies, reporting 110 outcomes. Effectiveness in reducing suicidal ideation was low (standardised mean difference −0.174, 95% CI −0.238 to −0.110). DBIs were significantly less effective against suicidal behaviours than against suicidal ideation, although still effective (standardised mean difference −0.059, 95% CI −0.087 to −0.032). Human involvement had no significant effect on effectiveness.

**Conclusions:**

Despite low effectiveness, DBIs might play a role in large-scale prevention efforts against suicidal ideation within a stepped care approach. Further, DBIs may be helpful in expanding mental health services in low- and middle-income countries with otherwise limited access to mental healthcare. Although the evidence for DBIs efficacy is well grounded, the technical and scientific evaluation of DBIs regarding their set up, functionality and components needs to be addressed in future studies.

Suicidal ideation and behaviours are a challenge for public health and service providers, given that annually 138 million people experience suicidal ideation, 20.7 million people attempt suicide^1^ and around 700 000 people die by suicide.^2^ Still, only 17–56% of persons experiencing suicidal ideation and behaviours receive treatment.^3^ These low treatment rates are linked to two main structural barriers: treatment cost and availability.^3^

## Barriers to treatment

Improving affordability and accessibility of treatment means to provide suicide-specific care in terms of tailored interventions according to the patient's stage of suicidal progression (e.g., pre-motivation, ideation only, plan/attempt^4^), rather than using a ‘one size fits all’ solution. A stepped care approach has been recommended to align with stages of suicidal progression. Accordingly, least-restrictive interventions at early stages such as suicidal ideation might involve, for example, telephone calls only, and most restrictive interventions at later stages might involve in-patient care.^5^ In this sense, early care should be available and affordable, lowering treatment barriers and, in turn, motivating individuals to seek help, who are otherwise hesitant to do so.^3^ Early interventions at the stage of suicidal ideation have been suggested to lower human suffering and prevent future suicides.^6^

## Distance-based interventions

Distance-based interventions (DBI) are least-restrictive treatments, in terms of local availability, affordability and available service hours. Underserviced areas can be supported by both tele-health and apps. Although in the short term the development and evaluation of apps and tele-health interventions are expensive, in the long run they are less expensive and less resource-intensive than individual psychotherapy, especially when a large number of people are treated.

During the past two decades, a number of randomised controlled trials (RCTs) examining DBIs have been published. Starting at the turn of the millennium, with studies using telephone calls^7^ and postcards,^8^ leading to crisis hotlines and email follow-ups.^9^ Recently the field has expanded to online programmes^10,11^ and, since the COVID-19 outbreak, increasingly to tele-health approaches.^12^ Several meta-analyses have been published on subsets of DBI.^13,14^

To give recommendations for future research and clinical practice, our meta-analysis differentiates between autonomous DBI (aDBI) (i.e. apps, online programmes) and human DBI (hDBI) (telephone calls, postcards, tele-health). Given that aDBIs have a superior scalability,^15^ it remains to be seen whether aDBI utilisation reduces effectiveness when compared with hDBI.

To draw practical conclusions, we asked three questions, implemented as moderation analyses: (a) are DBIs effective against suicidal ideation and/or against suicidal behaviours, (b) are interventions’ effects stable over time and (c) is the effectiveness of interventions independent from the primary study control groups (treatment as usual (TAU)/attention placebo/waitlist)?

## Method

The systematic search followed the Preferred Reporting Items for Systematic Reviews and Meta-Analyses guidelines^16^ and was pre-registered on Prospero under the pre-registration number CRD42020218791.

Search strings were defined with repeated searches combining MeSH terms relating to suicide prevention or intervention, with the intervention types (e.g.) letter, app, web-based or distance. The resulting search string was tested and refined with two related meta-analyses, one on hDBI^13^ and one on aDBI^14^ (see Appendix for final strings).

Once search strings were established, the first hundred search results of Web of Science were examined together by authors J.S. and K.R., establishing a common degree of understanding. Afterward, both authors independently searched Web of Science, Scopus and PubMed; systematic searches were last updated in December 2021. Cohen's kappa between both authors was 0.806.

### Inclusion and exclusion criteria

All peer-reviewed RCT studies were included, which investigated any form of DBI with at least one primary outcome being suicidal ideation and/or behaviours, such as suicidal planning, suicide attempts and death by suicide. Face-to-face meetings were allowed if these were not part of the intervention, i.e. for informing, testing or screening purposes.

All suicidal ideation and behaviour outcomes of applicable studies were coded, excluding combined outcome measures such as the total score of Suicidal Behaviours Questionnaire Revised, which sums lifetime ideation and behaviours in a total score.

### Data extraction and coding

Data was coded independently by two authors (J.S. and K.R.). If possible, non-imputed results were coded. The following variables were extracted: author, year, control group of study, country of study, sample type, sample size, intervention type, gender ratio, mean age, mean age (s.d.), outcome name (e.g. suicidal ideation), intervention duration in weeks, participant attrition rate, follow-up time, standardised mean difference (SMD) and variance of SMD. In addition, all outcomes were coded for the moderation analysis into subgroups (see Table 1).

**Table 1:** Outcome allocation to moderator analyses

Moderator group	Outcome name
Behaviours	Suicide, suicide attempts, self-harming behaviours
Ideation	Suicidal thoughts, suicidal ideation, suicide plans
Human involved	Telephone calls, (video call based) cognitive–behavioural treatment, personalised letters or personalised e-mails
Autonomous	Applications, websites, non-individualised letters or non-individualised e-mails
Treatment as usual	Treatment as usual, enhanced treatment as usual, intensive case monitoring
Attention placebo	Attention placebo, control article, journaling, attention control, control programme, body positivity images
Waitlist	No contact, reminder letter at the end, waitlist, no interventions

The authors compared the finalised coding sheets, discussed differences and re-coded affected studies until a unanimous result was achieved. Risk of bias was assessed using the Risk of Bias 2 (RoB-2),^17^ and Trim and Fill was used as the publication bias detection method.^18,19^

### Statistical method

To incorporate all outcomes of interest we used a three-level meta-analysis (multi-level meta-analysis; MLM),^20,21^ which directly assesses research heterogeneity between different outcomes.^22^ In addition, we applied robust variance estimation (RVE),^23,24^ which returns valid confidence intervals in the presence of complex data dependencies.^25^ The use of both MLM and RVE in a mixed model yields higher robustness, superior precision and minimal outcome selection bias, compared with MLM or RVE only models or standard meta-analysis.^26^ Models were fitted with restricted maximum likelihood estimation and RVE correction was based on Pustejovsky and Tipton.^27^

Calculations were done in R (Version 4.2.0, MacOS: https://www.r-project.org/),^28^ using the package *metafor* for the three-level model^29^ and the package *clubSandwich*^30^ for the RVE correction. All data needed for full reproducibility, as well as any deviations from the pre-registered report, are publicly available on GitHub (https://github.com/jim-schmeckenbecher/Distance-based-Interventions).

### Sensitivity analysis and bias

Given that non-suicidal self-injury (NSSI)^31^ and suicidal behaviours^32^ differ qualitatively, we employed two sensitivity analyses: (a) including NSSI as an outcome and (b) excluding suicide deaths as an outcome. RoB-2^17^ and Trim and Fill were used to assess risk of bias and publication bias detection, respectively.^18,19^

## Results

**Fig. 1:**
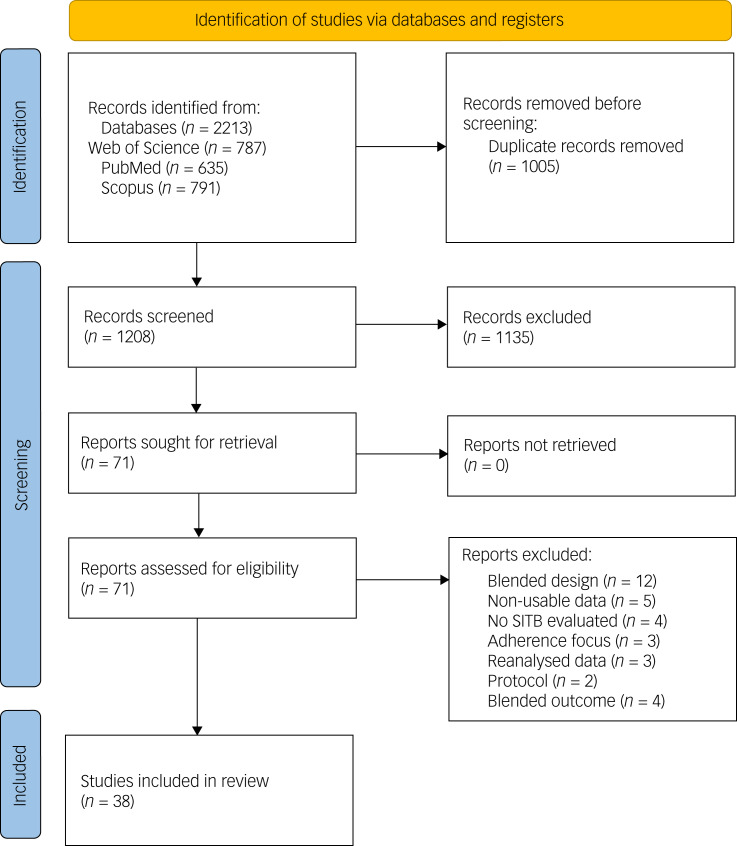
Flow chart of all studies.

We identified 2213 papers in the databases (see Fig. 1), and 35 independent RCT trials were included in the analysis. The difference between included studies in the flow chart and reported independent RCT trials is explained by follow-up studies that used the same sample as their parent study. These study pairs have been treated as a single RCT. Further, one study included three statistically independent RCTs.^11^

Overall, 110 outcomes emerged, with 65 outcomes of the category suicidal ideation, including planning, and 45 outcomes of the category behaviours, including studies examining mostly self-harm and suicide attempts.

Twenty outcomes were found for hDBIs and 90 outcomes were found for aDBIs. The median duration of studies was 26.00 weeks, with a range of 0.14–782 weeks. The median time between post-intervention measures and follow-up measures was 17 weeks, with a range of 0.86–522 weeks. Finally, 52 outcomes were based on TAU group comparisons, 14 outcomes were based on waitlist control groups and 44 outcomes were based on attention placebo control groups. The median attrition rate was 17.00%, with a maximum of 64.50% and a minimum of 0%.

### Sample characteristics

In total, 11 158 participants were included at post-intervention and 9201 at follow-up. Out of all participants, 64.43% were female and on average 31.87 (s.d. 10.01) years old. The youngest reported mean sample age was 14.70 (s.d. 1.46) years, the oldest mean sample age was 51.00 (s.d. 11.39) years.

Out of 35 studies, most data was retrieved from Westernised educated industrialised democracies, predominantly the USA (*k =* 10), followed by Australia (*k* = 9). Five studies emerged from non-Westernised educated industrialised democracies.

### Main analysis

DBIs were effective in reducing suicidal ideation and behaviours (SMD = −0.121, 95% CI −0.166 to −0.077); heterogeneity was significant at *Q* = 154.658 (d.f. = 109, *P =* 0.003).

**Fig. 2:**
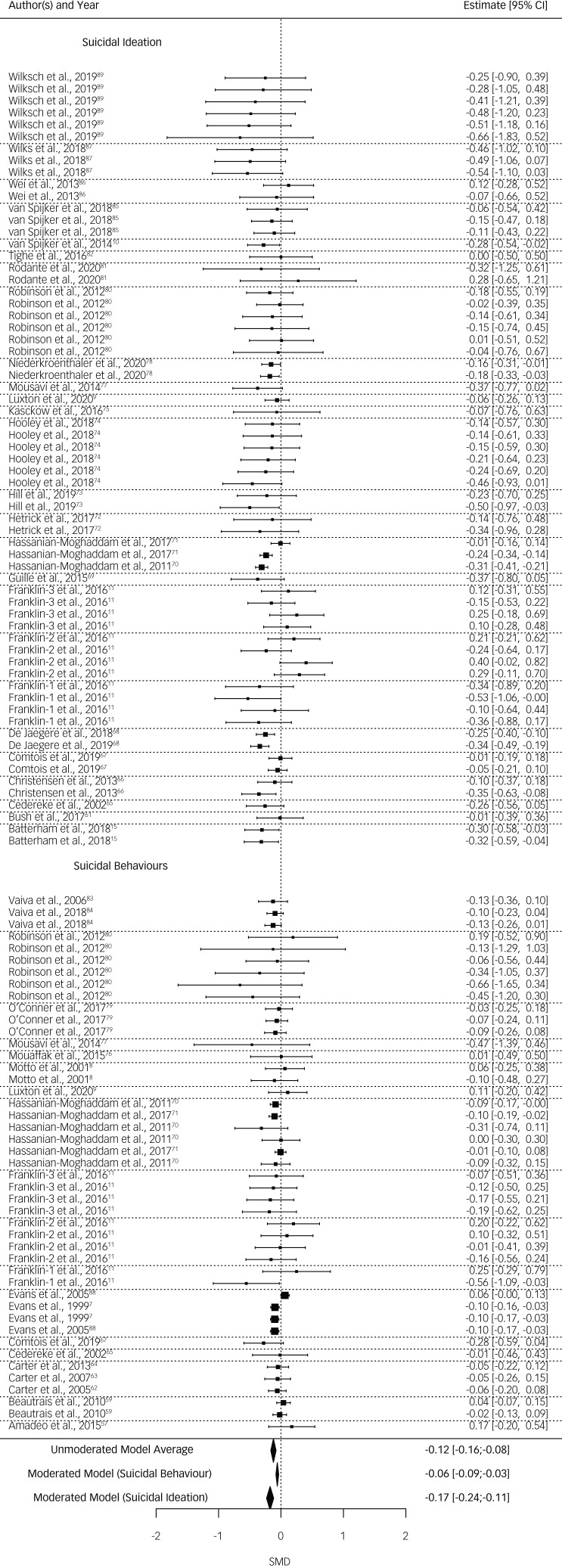
Forest plot, ordered after behaviour and ideation subsets. Dotted lines represent study-level dependencies.

DBI are more effective against suicidal ideation than suicidal behaviours (SMD *=* −0.115, 95% CI −0.181 to −0.047, *P* = 0.004). The average effectiveness against suicidal ideation was SMD *=* −0.174 (95% CI −0.238 to −0.110), whereas suicidal behaviour was significantly lower at SMD *=* −0.059 (95% CI −0.087 to −0.032). Heterogeneity was non-significant at *Q* = 118.457 (d.f. = 108, *P =* 0.231; see Fig. 2).

Because of the small number of studies using a waitlist condition, the comparison of waitlist and attention placebo groups were not trustworthy according to their profile likelihood plots.^33^ Therefore, waitlist and attention placebo were combined into one group and compared with TAU.

Effectiveness of the DBI was significantly dependent on the control group. DBI versus waitlist and attention placebo (SMD *=* −0.175, 95% CI −0.235 to −0.114) were significantly more effective in reducing suicidal ideation or behaviours (SMD *=* 0.097, 95% CI 0.018–0.176, *P* = 0.017) than DBI versus TAU (SMD *=* −0.078, 95% CI −0.129 to −0.027). Heterogeneity remained significant at *Q* = 139.797 (d.f. = 108, *P =* 0.021).

**Fig. 3:**
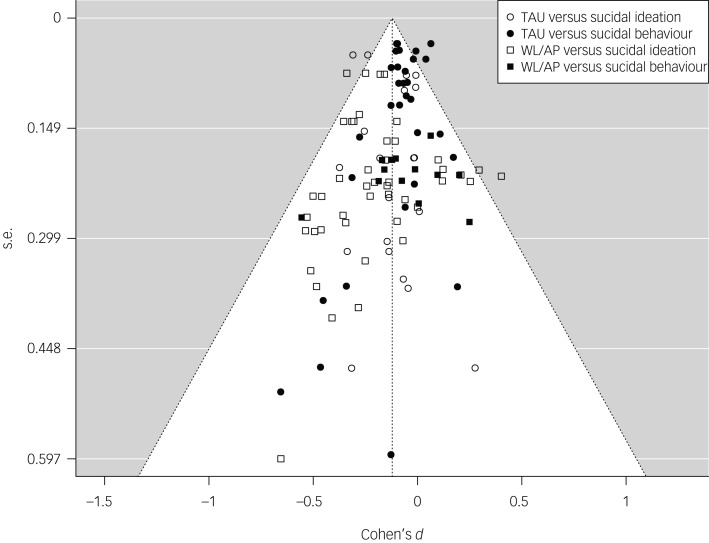
Funnel plot, including all outcomes according to moderator type. TAU, treatment as usual; WL/AP, waitlist and attention placebo.

Possible covariance of control group type (i.e. TAU versus waitlist and attention placebo) and outcome type (i.e. ideation versus behaviours) was investigated. As suicidal ideation and suicidal behaviours were unevenly distributed between studies using different control-group types (see Fig. 3), an exploratory analysis including both moderators was implemented. When including both moderators, the difference between control groups became non-significant (SMD *=* 0.041, 95% CI −0.059 to 0.140, *P* = 0.390), but suicidal behaviours and suicidal ideation remained a significant moderator (SMD *=* −0.097, 95% CI −0.187 to −0.006, *P* = 0.040) in favour of suicidal ideation. Heterogeneity was not significant at *Q* = 118.037 (d.f. = 107, *P =* 0.219).

Effectiveness of the DBI decreased non-significantly between time points (SMD *=* 0.028, 95% CI −0.026 to 0.082, *P =* 0.271). Heterogeneity was significant at *Q* = 152.451 (d.f. = 108, *P =* 0.003).

aDBI and hDBI did not differ significantly in effectiveness (SMD *=* −0.061, 95% CI −0.142 to 0.019, *P =* 0.1213). Heterogeneity was significant at *Q* = 147.094 (d.f. = 108, *P =* 0.007).

### Sensitivity analysis and bias

Both inclusion of NSSI outcomes (*o* = 10) and exclusion of suicide death outcomes (*o* = 4) had negligible effects on the subgroup of suicidal behaviour outcomes. The inclusion of NSSI did not change the effectiveness of DBIs (SMD_−NSSI_ = −0.059 compared with SMD_+NSSI_ = −0.060), and exclusion of suicide death outcomes did not change the effectiveness (SMD_+suicide death_ = −0.059 compared with SMD_−suicide death_ = −0.055). It has to be noted that only Franklin et al^11^ reported NSSI measures in a study aimed at reducing suicidal behaviour and suicidal ideation as a primary outcome.

Risk of bias of all independent studies was mixed; see the online supplementary material (https://github.com/jim-schmeckenbecher/Distance-Based-Interventions) for a full RoB-2 assessment. According to Trim and Fill, no publication bias was observed.

## Discussion

In this meta-analysis, we examined the effectiveness of DBIs reducing suicidal ideation and behaviour. The quality of evidence was good, including a substantial number of high- and medium-quality studies and no observed publication bias. On average, DBIs reduced both suicidal ideation and suicidal behaviour.

### Contextualising results with other meta-analyses

We contextualise our findings using meta-analyses of psychotherapeutic face-to-face interventions for suicidal ideation and/or behaviour. Because it can be argued that such a comparison is biased because each meta-analysis has different inclusion criteria, designs and underlying assumptions, it nevertheless offers a rough estimation of the effect sizes to be expected from a suicide intervention. To maximise comparability, we only include meta-analyses on psychotherapeutic face-to-face interventions, researched by RCT studies, using TAU, waitlist or attention placebo control groups, published in the past decade.

We searched Web of Science using the search term: ‘“suic*” AND “therap*” AND (meta-analys* OR meta analys*)’. Out of 350 face-to-face intervention results, nine meta-analyses with the primary outcome suicidal ideation or behaviours were found.^34–42^ One^39^ did not exclude particular populations or therapeutic approaches.

#### Effectiveness of DBIs for suicidal behaviours

We showed that DBI significantly reduced suicidal behaviours (SMD = −0.059, 95% CI −0.087 to −0.032). When comparing the results of our meta-analysis on DBI with the results of the nine meta-analyses on face-to-face interventions, we found three meta-analyses with significantly higher results, whereas six meta-analyses reported non-significant differences. Two of the three meta-analyses reporting significantly higher results were comprised of adolescent samples^35,36^ and used ‘self-harm’ as an outcome. Compared to our DBI effect sizes, both show significantly higher reductions in ‘self-harm’ through dialectical behavioural therapy. However, the effects of dialectical behavioural therapy were only found post-treatment^35,36^ and did not outlast the 3-month follow-up.^35^ In addition, eclectic therapy led to a significantly higher effectiveness at post-treatment compared with DBIs, but also to an increase in ‘self-harm’ at follow-up.^35^ The third meta-analysis reporting significantly stronger results than our DBI effect sizes included only psychoanalytic approaches^38^ and used ‘suicide attempts’ and ‘self-harm’ as outcomes. Psychoanalytic approaches reduced ‘self-harm’ significantly at 6-month follow-up, although their effects were non-significant at 12-month follow-up. All these meta-analyses predominantly used TAU as control groups.

The above comparisons of the effect of face-to-face interventions with the effectiveness of DBIs shows strong ambiguity, it seems that face-to-face interventions show time effects and some seem even harmful.^35^ We argue that these meta-analyses on face-to-face interventions suggest that research is often too underpowered to evaluate the potential superiority of different therapeutic approaches with statistical certainty. For this purpose, confidence intervals inform us about the clinical potential of an intervention. For example, the effect size presented by Briggs et al,^38^ who examined the effects of psychoanalytic therapies against ‘suicide attempt episodes at 12 months follow-up’, can be transformed into a number needed to treat (NNT) of 7.577 (95% CI infinite to 3.605). This means the intervention could be ineffective (infinite NNT), or could help up to 1 in 3.6 patients. Accordingly, the effect sizes of DBIs, transformed into NNT *=* 30.049 (95% CI 55.393–20.386), mean that suicidal behaviours will be reduced with a 95% certainty in at least 1 of 55 patients, and at best, every twentieth patient will benefit from a DBI. The small but statistically certain effect of DBIs against suicidal behaviour supports the conclusion that DBIs as a least-restrictive intervention at an early stage of progression^5^ could be used as an add-on intervention, within the scope of suicide preventive strategies. However, the effect sizes do not allow us to recommend DBI as a standalone intervention for suicidal behaviour. But, given the evidence, DBI might remain the only possible option in circumstances where no other intervention is available, and gives the possibility to reach suicidal persons otherwise not engaged in treatment.

#### Effectiveness of DBIs for suicidal ideation

According to our results, suicidal ideation is significantly reduced by DBI (SMD = −0.174, 95% CI −0.238 to −0.110). In comparison with the only meta-analysis examining face-to-face interventions and not differentiating between therapeutic approaches or populations (adults, adolescents, diagnosis), face-to-face interventions were more effective against suicidal ideation than DBIs.^39^ However, some meta-analyses focusing on different therapeutic approaches^34–36^ reported comparable results to ours investigating the use of DBIs. However, we assume that this non-significance was the result of lower power compared with Hetrick et al.^39^

In contrast to our recommendations for patients with suicidal behaviour, we see a role for DBIs in the prevention of suicidal ideation. DBIs can potentially be utilised in all domains of prevention (universal, selective, indicated and treatment).^43^ They would allow to reach at least part of the 138 million people facing suicidal ideation each year,^1^ and to engage them in a low-cost, evidence-based and least-restrictive intervention allowing them to initiate a transition into regular care at psychiatric services where available.

### Research recommendations

According to our results, aDBIs were as effective as hDBIs. This finding highlights the potential of aDBIs, especially given their good scalability,^15^ making large-scale and replication studies easier to implement. However, it still remains unclear which aDBI components are most effective, or how aDBIs should be designed to achieve best results. Similarly, some assumptions about aDBIs for mental health lack evidence, such as the assumed superiority of their 24 h availability.^44^ In contrast, the best evidence derived from meta-analytic studies support cost-effectiveness, acceptability and satisfaction of DBI by users. Notably, this evidence relates to mental health interventions in general,^44,45^ and needs to be validated for DBIs with a suicide prevention focus. Therefore and in contrast to the evidence of therapeutic efficacy, the technical development and scientific validation of aDBI components is still in its infancy and needs to be addressed through future research.

### Implications for clinical practice

The therapeutic efficacy of DBIs is supported by our evidence and might play a role in different levels of suicide-specific care. As mentioned, Jobes et al^5^ outline five levels of intervention in a stepped care model for suicide, ranging from least-restrictive interventions like telephone (level 1), brief interventions (level 2), out-patient care (level 3) and partial hospital admission (level 4); and the most restrictive interventions like in-patient care/full hospital admission (level 5). Given the presented evidence, we see a role for DBIs to supplement the lower spectrum of the stepped care model, namely (human-based) ‘telephone interventions and follow-ups’ and ‘brief interventions and follow-ups’.

In line with the meta-analyses on face-to-face interventions, our results show that DBIs are more effective in treating suicidal ideation than suicidal behaviours. These findings support the argument of providing help as early as possible, preferably before suicide behaviours even emerge.^46^ It is well-known that the density of mental health service providers is geographically unevenly distributed.^47,48^ This problem was compounded by the ongoing COVID-19 pandemic, which caused lockdowns and associated barriers for face-to-face therapies and raised the acceptability of tele-therapy. DBI may be able to mitigate the growing need for distance-based treatment. Moreover, the reality of psychiatric treatment includes high costs for the individual or the health insurance,^49^ depending on insurance coverage, as well as long waiting times for patients.^50^ In both cases, DBI can be an intermediate solution bridging waiting times and reducing costs and human suffering. Finally, low-cost aDBI can help to expand mental health services, especially in low- and middle-income countries, as called for by Chisholm et al,^51^ and as such, contribute to the sustainable development goals for mental health set by the United Nations.^52^ As evidence for DBI continues to emerge and tele-health and other forms of interventions develop, we recommend establishing suicide-specific training frameworks^53,54^ that incorporate DBI as an adjunct to standard healthcare services and professional training.

### Limitations

Given our approach, some potential limitations should be noted. First, it could be seen as a limitation that most studies included in this meta-analysis are already covered by previously published meta-analyses.^13,14^ However, these previously published meta-analyses used a methodological approach (Hedges–Olkin meta-analysis) with which only one data point per independent data-set can be included; in contrast, the method used by us allows for the inclusion of all relevant data.^21^ Utilising all relevant data has multiple advantages, foremost higher precision and less risk of bias. Further, it allows important moderator analyses to be implemented in one model, meaning evidence is weighted according to its informational value. In contrast, previous meta-analyses often had to use independent subgroup analyses, which have lower precision and only allow for indirect comparisons. Bias risk, selecting one outcome per independent analysis, can introduce a selection bias because it works under the assumption that the chosen outcome is representative of all other outcomes. Based on these points and the fact that we updated and broadened the systematic searches of previous meta-analyses, the current meta-analysis substantially adds to the research field.

Despite the above-stated advantages of the chosen method, this method also introduces two potential limitations. First, the employed method requires more studies to reach adequate power, risking underpowered results.^55^ Still, our results were trustworthy and adequately powered, according to profile likelihood plots^33^ and the degrees of freedom form RVE correction.^27^ Second, RVE-corrected models do not include heterogeneity estimations. Therefore, heterogeneity estimations of the multi-level model were reported, as *Q-*test results are not biased by dependency and therefore statistically valid and of adequate power.^56^

In conclusion, the presented results are based on 35 published, peer-reviewed, independent RCT trials on DBIs. With adequate power, no indication for publication bias and manageable heterogeneity, the results suggest that DBIs, particularly aDBIs, are an effective adjunct intervention to face-to-face treatment of individuals with suicidal ideation, and especially in situations where availability of face-to-face treatments is limited.

## Data Availability

All data is available at GitHub: https://github.com/jim-schmeckenbecher/Distance-based-Interventions.

## Supplementary material

For supplementary material accompanying this paper visit http://doi.org/10.1192/bjo.2022.526. click here to view supplementary material

## Appendix

### Search strings

Note: Search string was adapted in Scopus adding: TITLE-ABS-KEY. This is implied when using WOS and Pubmed. As in WOS and Pubmed, Title, Abstract and Keywords were also searched; both strings are in practice identical.

### For WOS and PubMed

((“Suic*” OR “Suicide prevention” OR “self harm*” OR “self poisoning*” OR “self injur*” OR “self mutilation”) AND (“telehealth” OR “postcard*” OR “onlin*”OR“Online Intervention” OR “Online Prevent*” OR “E Intervention” OR “E-Intervention” OR “E Prevention” OR “Electronic Intervention” OR “Electronic Prevention” OR “Mobile Intervention” OR “Mobile Prevention” OR “Web-Based*” OR “Web Based*” OR “Online Support” OR “E Therapy” OR “e-mail*” OR “e mail*” OR “App” OR “Apps” OR “App-Assis*” OR “mobile-App” OR “mobile health intervention” OR “telephone” OR “phone based” OR “letter*”) AND (RCT OR Random*)).

### Scopus

(TITLE-ABS-KEY ((“Suic*” OR “Suicide prevention” OR “self harm*” OR “self poisoning*” OR “self injur*” OR “self mutilation”) AND (“telehealth” OR “postcard*” OR “onlin*” OR “Online Intervention” OR “Online Prevent*” OR “E Intervention” OR “EIntervention” OR “E Prevention” OR “Electronic Intervention” OR “Electronic Prevention” OR “Mobile Intervention” OR “Mobile Prevention” OR “Web-Based*” OR “Web Based*” OR “Online Support” OR “E Therapy” OR “e-mail*” OR “e mail*” OR “App” OR “Apps” OR “App-Assis*” OR “mobile-App” OR “mobile health intervention” OR “telephone” OR “phone based” OR “letter*”) AND (rct OR random*)))
