# Supplementary material for: Effectiveness of distance-based suicide interventions: multi-level meta-analysis and systematic review
Source: BJPsych Open. 2022 Jul 21;8(4):e140. doi: 10.1192/bjo.2022.526 (PMC9345623; doi:10.1192/bjo.2022.526)
Supplement: Supplementary file 1 [file S2056472422005269sup001.pdf]

**Study ID**	**D1**	**D2**	**D3**	**D4**	**D5**	**Overall**
Amadéo et al., (2015)	+	!	+	-	!	-
Batterham et al., (2018)	+	+	+	+	+	+
Beautrais et al., (2010)	-	+	+	+	+	-
Bush et al., (2017)	+	!	+	!	!	!
Carter et al., (2005)	+	+	+	+	+	+
Cederke et al., (2002)	+	!	+	+	+	!
Christensen et al., (2013)	+	+	+	-	+	-
Comtois et al., (2019)	+	!	+	+	+	!
De Jaegere et al., (2019)	+	+	-	+	+	-
Evans et al., (1999)	+	+	+	+	!	!
Franklin et al., (2016)	!	+	!	-	+	-
Guille et al., (2015)	+	+	+	!	!	!
Hassanian-Moghaddam et al., (2011)	+	+	+	+	+	+
Hetrick et al., (2017)	!	-	-	+	+	-
Hill et al., (2019)	+	!	+	-	!	-
Hooley et al., (2018)	+	!	+	+	+	!
Kasckow et al., (2016)	!	-	+	-	!	-
Luxton et al., (2020)	-	+	+	+	+	-
Motto et al., (2001)	!	+	+	+	!	!
Mouaffak et al., (2015)	+	!	+	+	!	!
Mousavi et al., (2001)	!	!	+	+	!	!
Niederkrotenthaler et al., (2020)	+	+	+	+	+	+
O`Conner et al., (2017)	+	+	+	+	+	+
Robinson et al., (2012)	!	!	+	-	+	-
Rodante et al., (2020)	!	+	+	!	!	!
Tighe et al., (2017)	+	!	!	-	+	-
van Spijker et al., (2014)	+	!	-	+	!	-
Van Spijker et al., (2018)	+	!	-	-	+	-
Vavia et al., (2006)	+	+	+	+	+	+
Vavia et al., (2018)	+	+	+	+	+	+
Wei et al., (2013)	!	+	!	+	+	!
Wilks et al., (2018)	!	!	+	+	+	!
Wilksch et al., (2019)	+	+	+	+	+	+

+ Low risk

! Some concerns

- High risk

D1 Randomisation process

D2 Deviations from the intended interventions

D3 Missing outcome data

D4 Measurement of the outcome

D5 Selection of the reported result

GRADE

Outcome	No. of independent studies	Study design	Risk of bias[1]	Inconsistency	Indirectness	Imprecision	Other considerations	Pooled SMD (95% CI) for subgroup[2]	Certainty
**Suicide Ideation**	27	RCT	serious	Not serious	Not serious	Not serious		-0.175 [-0.24;-0.107]	⊕⊕⊕○ MODERATE
**Suicide Attempts**	8	RCT	Not serious	Not serious	Not serious	Not serious		-0.07[-0.131;-0.014]	⊕⊕⊕⊕ High
**Suicide**	1	RCT	Not serious	Not serious	Not serious	serious	One follow up study included; with large sample and long follow up period	NA	⊕⊕⊕○ MODERATE
**Deliberate Self Harm**	5	RCT	serious	Not serious	Not serious	Not serious		-0.06 [-0.11; -0.01]	⊕⊕⊕○ MODERATE
**Deliberate Self Poising**	1	RCT	Not serious	Not serious	Not serious	serious		NA	⊕⊕⊕○ MODERATE
**Suicide Planning**	6	RCT	serious	Not serious	Not serious	Not serious		-0.1829 [-0.3567; -0.0091]	⊕⊕⊕○ MODERATE
**Suicidal Thoughts**	1	RCT	serious	NA	Not serious	serious		NA	⊕⊕○○ Low
**Suicidal Event**	1	RCT	Not serious	NA	Not serious	Not serious	One follow up study, with large sample	NA	⊕⊕⊕○ MODERATE
**Self Harm**	3	RCT	Not serious	Not serious	Not serious	Not serious		-0.01 [-0.067;0.049]	⊕⊕⊕⊕ High
**NSSI**	3	RCT	serious	Not serious	Not serious	serious	Only in one study with 3 independent sub-samples, with very small sample sizes	-0.095 [-0.31; 0.119]	⊕⊕○○ Low
**Suicidal Behaviour**	1	RCT	serious	NA	Not serious	serious		NA	⊕⊕○○ Low

1 Estimated based on RoB-II average: Which was estimated conservatively.
2 Due to Data Limitations, Analysis were conducted using Hedges Olkin Analysis on study level aggregated Data.

Table 2. *Overview of independent studies and their sample characteristics*

Authors	N (TG/ CG)	Age M(SD)	Female %	Country	Included Outcomes	Follow-up	Intervention Type
Amadeo et al. (2015)	190 (100/90)	32	64	PYF	suicide attempts, completed suicides	NA	Phone calls
Batterham (2018)	194 (118/76)	NA	86	AUS	Suicide ideation	At 12 weeks	Online module program
Beautrais et al. (2010)	327 (153/174)	33.8	66	NZL	deliberate self-harm	NA	6 Postcards
Bush et al. (2017)	118 (58/60)	47	32	USA	Suicide ideation	NA	Smartphone App
Carter et al. (2005)	772 (378/394)	38.7	68	AUS	deliberate self-poisoning	2 and 5 years later (Carter et al., 2007; Carter et al., 2013)	8 postcards
Cedereke et al. (2002)	216 (107/109)	41	66	SWE	suicide ideation, suicide attempts	After 17 weeks	Telephone calls
Christensen et al. (2013)	155 (TG1=38; TG2=45; TG3=37; /35)	41.49	81.9	AUS	suicide ideation	After 24 weeks	Web-based Cognitive Behaviour Therapy
Comtois et al. (2019)	658 (329/328)	25.2	18	USA	suicide ideation, suicide attempts	NA	11 Text Messages
De Jaegere et al. (2019)	724	59.4	35.7	BEL	suicidal thoughts	After 6 weeks	Unguided

Authors	N (TG/ CG)	Age M(SD)	Female %	Country	Included Outcomes	Follow-up	Intervention Type
	(365/359)						online self-help intervention
Evans et al. (1999)	827 (417/410)	33.5	55	GBR	deliberate self - harm	26 weeks later (Evans et al., 2005	crisis card & 24h telephone support
Franklin et al. (2016)				USA & EU	Non suicidal self-injury, deliberate self- harm, suicidal behavior, Suicide ideation, suicide plans	2 weeks	Mobile App
Study 1	114 (55/59)	23.02	80.77				
Study 2	131 (62/69)	22.9	74.1				
Study 3	163 (75/84)	24.5	58.6				
Guille et al. (2015)	199 (100/99)	25.2	49.3	USA	Suicide ideation	NA	Online CBT
Hassanian-Moghaddam et al. (2011)	2113 (1043/1070)	24.1	66.4	IRN	suicide ideation , deliberate self-poisoning, self – harm, suicide attempts,	52 weeks later (Hassanian-Moghaddam et al., 2015)	Postcards
Hetrick et al. (2017)	50 (26/24)	14.7	82	AUS	suicide ideation	12 weeks later	Online CBT
Hill & Pettit (2019)	80 (40/40)	16.67	68.8	USA	suicide ideation	6 weeks	Online program
Hooley et al. (2018)	144 (TG1= 49 TG2=49	25.63	85.4	USA	suicide ideation, suicide plans	4 and 8 weeks	Online daily diary

Authors	N (TG/ CG)	Age M(SD)	Female %	Country	Included Outcomes	Follow-up	Intervention Type
	/46)						
Kasckow et al. (2016)	32 (16/16)	51.1	6	USA	suicide ideation	NA	Health Buddy System
Luxton et al. (2019)	1318 (652/666)	32	24	USA	suicide ideation Suicide mortality	NA	13 caring emails
Motto & Bostrom (2001)	843 (389/454)	34	56	USA	suicide rates	522 weeks	Short letters
Mouaffak et al. (2015)	320 (160/160)	39	73.5	FRA	suicide attempts	NA	resource card, phone calls
Mousavi et al. (2014)	139 (69/70)	NA	64	IRN	Suicide ideation Suicide reattempts	NA	7 Telephone calls
Niederkrotenthaler et al. (2020)	266 (TG1=86 TG2=90 /90)	34	73	AUT	Suicide risk	1 week later	Newspaper articles
O`Connor et al. (2017)	518 (259/259)	36	63	GBR	self – harm	NA	volitional helpsheet
Robinson et al. (2012)	165 (81/83)	18.6	62	AUS	Suicide ideation suicide-related behavior, deliberate self – harm	26 weeks later	12 postcards
Rodante et al. (2020)	18 (9/9)	30	81	ARG	suicide ideation, suicide plans	4 weeks later	Mobile App-DBT
Tighe et al. (2017)	61	26	64	AUS	Suicide ideation	NA	acceptance-

Authors	N (TG/ CG)	Age M(SD)	Female %	Country	Included Outcomes	Follow-up	Intervention Type
	(31/30)						based therapy App
Vaiva et al. (2006)	605 (TG1=147 TG2=146 /312)	36	73.2	FRA	deliberate self-poisoning, suicide attempts, death by suicide	9 oder 11 mo	Telephone calls
Vaiva et al. (2018)	949 (461/488)	38.7	64.8	FRA	Suicide reattempts	NA	Postcards and Telephone
van Spijker et al. (2014)	236 (116/120)	41	66.1	NLD	suicidal thoughts	NA	Online module- based program
van Spijker et al. (2018)	418 (207/211)	41	77.3.	AUS	suicidal thoughts	52 weeks	Online module- based program
Wei et al. (2012)	239 (TG1 =82 TG2= 80 CG=77)	33	76	PRC	Suicide attempts	40 weeks	12 telephone calls
Wilks et al. (2018)	59 (30/29)	38	69.5.	AUS	Suicide ideation	At 5 and 9 weeks	Online DBT skills training
Wilksch et al. (2019)	316 (122/194)	21	100	AUS & NZL	suicide ideation	26 and 52 weeks	Online module- based program
